# Effects of different psychosocial interventions on death anxiety in patients: a network meta-analysis of randomized controlled trials

**DOI:** 10.3389/fpsyg.2024.1362127

**Published:** 2024-03-18

**Authors:** Jinhong Lu, Youzhuan Yang, Haiyun Chen, Hongchao Ma, Yulei Tan

**Affiliations:** Department of General Surgery, Zhujiang Hospital, Southern Medical University, Guangzhou, China

**Keywords:** effects, psychosocial interventions, death anxiety, network meta-analysis, randomized controlled trials

## Abstract

**Objective:**

This research intended to assess and compare influence of psychosocial interventions in death anxiety in patients, providing evidence-based guidance for both patients and healthcare providers.

**Design:**

The present study exclusively gathered randomized controlled trials by comprehensively searching across multiple databases, comprising of PubMed, Embase, Cochrane Library, Web of Science, and Scopus. The methodological quality of the enrolled studies involved in the analysis was assessed using the Cochrane bias risk assessment tool, and data analysis was performed utilizing appropriate software.

**Results:**

This research, encompassing 15 randomized controlled trials with a cumulative sample size of 926 patients, spanned from the earliest possible date to December 2023. The findings of network meta-analysis unveiled that the Rational-Emotive Hospice Care Therapy significantly reduced death anxiety among patients (Sequentially Updated Cumulative Ranking Analysis: 100%).

**Conclusion:**

The ranking plot of the network suggested that the rational-emotive hospice care therapy exhibited superior efficacy as a psychological treatment for reducing the death anxiety of patients.

**Systematic review registration**: [https://clinicaltrials.gov/], identifier: [CRD42023484767].

## 1 Introduction

Death anxiety (DA), while often considered an anxiety disorder, is more precisely defined as an ongoing and excessive concern about dying (Sharif Nia et al., 2020). It is essential, however, to differentiate between a pathological fear of death and a normal level of concern about mortality. According to terror management theory (Arndt et al., 2005), the awareness of mortality is a universal condition that can evoke a spectrum of responses, from healthy emotional reactions to debilitating fear. Only when the anxiety becomes excessive, does it associate with clinical disorders, highlighting the nuanced nature of death anxiety (Lane et al., 2015). Additionally, the interchangeability of death fear and anxiety in literature often blurs the distinction between these constructs. While many scholars use these terms synonymously, it is important to note that emotion literature typically differentiates between fear, a more immediate and specific reaction, and anxiety, a broader and more diffuse apprehension (Lang and McTeague, 2009). This manuscript endeavors to clarify this distinction by examining the broader construct of death anxiety, which includes a range of fears associated with death and dying. Among individuals facing illness, the experience of death anxiety is common and considered a natural response. Notably, a 2018 study in Iran revealed that more than 90% of heart failure patients exhibit moderate to severe DA (Asgari et al., 2018). Similarly, among patients with various cancers in advanced stages, 32% report experiencing DA (Neel et al., 2015). The COVID-19 pandemic has further exacerbated this issue, with 35.5% of Chinese patients experiencing severe DA (Zeng et al., 2021). Death anxiety is closely associated with adverse psychological outcomes, including depression, dependency, and fear of pain and dying (Lo et al., 2010). This fear of death transcends specific diagnoses and can contribute to the progression and persistence of various chronic mental health issues. Meanwhile, for patients who have successfully undergone early-stage treatment, DA may serve as a catalyst for relapse or the onset of new diseases (Menzies and Menzies, 2023). Acknowledging the complexity of death anxiety, a variety of therapeutic interventions have emerged, each based on distinct theoretical underpinnings. For instance, rational-emotive hospice care therapy is based on the principles of rational emotive behavior therapy (REBT), targeting irrational beliefs about death to reduce anxiety. In contrast, logotherapy emphasizes finding meaning in life, even in the face of death, as a way to mitigate the existential distress associated with death anxiety. Cognitive-behavioral therapy, acceptance and commitment therapy, and other interventions similarly aim to address the psychological factors contributing to death anxiety, offering patients a variety of coping mechanisms. This research seeks to explore and compare these interventions, providing evidence-based insights into their effectiveness in reducing death anxiety among patients. Several therapeutic approaches, such as spiritual care, group logotherapy, acceptance and commitment therapy, cognitive-behavioral therapy, and rational-emotive hospice care therapy have been empirically proven to effectively alleviate the experience of DA in patients (Onyechi et al., 2016; Saeedi et al., 2016; Nabipour et al., 2018; Emafti et al., 2019; Farahi and Khalatbari, 2019; Amini et al., 2020; Akbari et al., 2021; Ameri et al., 2021; Bahar et al., 2021; Sahebanmaleki et al., 2021; Ahmadi et al., 2022; Fadhil et al., 2022; Moradi et al., 2022; Saki et al., 2022; Heidary et al., 2023). This meta-analysis exclusively includes randomized controlled trials (RCTs) to ensure the highest level of evidence and minimize bias. The selection of patients for this review was based on the inclusion criteria aimed at capturing the broad spectrum of individuals experiencing death anxiety, without restriction to specific patient types, to enhance the generalizability of our findings.

Rational-emotive hospice care therapy, derived from the rational emotive behavior therapy (REBT) theory, presents a family-centered palliative treatment approach and/or counseling intervention for end-of-life care, specifically designed to address the identified issues of patients with all types of cancers and their family caregivers, including problem hypothesis, death anxiety, and psychological distress (Onyechi et al., 2016). In a randomized controlled trial (RCT) conducted by Onyechi in Nigeria with 32 cancer patients, the 10-week rational-emotive hospice care therapy intervention demonstrated success in reducing approximately two-thirds of death anxiety in the participants with various cancers (Onyechi et al., 2016). Logotherapy, a psychotherapeutic method focused on enhancing a patient’s sense of meaning in life, has shown positive influence on quality of life, life satisfaction, and symptom alleviation of patients in various research studies (Zimmermann et al., 2014, 2016). Adele conducted an RCT in Iran and demonstrated that after 10 weeks of logotherapy treatment, patients experienced a 1/5 reduction in their level of DA (Bahar et al., 2021). Cognitive-behavioral therapy is a psychological therapy that can alleviate the psychological burden of individual diseases and help patients recover normal functions (Enright, 1997). An research by Mohsen revealed that cognitive-behavioral therapy remarkably diminishes DA and depression in patients with heart failure (Moradi et al., 2022).

Various psychological therapies possess unique characteristics and may yield diverse effects on patients grappling with DA. Most previous meta-analyses predominantly focused on DA, concurrently examining the acceptance level of death, a gap persists in the literature, with no systematic review exclusively addressing the fear of individual death among patients. Consequently, evidence-based recommendations for the most suitable psychosocial intervention to alleviate DA in individuals remain scarce. Given this context, identifying a psychosocial intervention specifically tailored for reducing DA becomes crucial, particularly when physicians are contemplating therapeutic options for patients with this concern. Therefore, network meta-analysis (NMA) was employed herein to compare a range of psychosocial interventions (spiritual care, group logotherapy, acceptance and commitment therapy, cognitive-behavioral therapy, logotherapy, rational-emotive hospice care therapy, spirituality therapy training, positive psychology group therapy, mindfulness based cognitive therapy, and white noise and Benson’s relaxation technique). Furthermore, it assessed the impact of these interventions on DA in patients comprehensively. By doing so, this research aims to furnish both patients and clinicians with a nuanced comprehension on impacts of these psychosocial interventions, ultimately contributing recommendations grounded in evidence for clinical practice.

Over the past decades, while there have been some systematic reviews addressing death anxiety (DA) among mixed non-clinical and clinical samples (Menzies and Menzies, 2023), these studies predominantly focused on the effects of psychotherapy on patients’ attitudes toward death, including death acceptance and DA. The theoretical contribution of our study lies in conducting the first novel network meta-analysis to compare the efficacy of various therapies in reducing patients’ DA, a topic not previously explored by scholars. Our findings specifically target the patients’ fear of death, introducing a new dimension of clinical significance. Furthermore, the analytical method employed in this study is highly rigorous. To assess the quality of the literature, each article was evaluated jointly by two scholars. In case of disagreement, the assessment process involved collaboration among three scholars. At the same time, we maximized the inclusion of literature, resulting in a generally high-quality final selection. Consequently, our evidence is notably comprehensive. Overall, this constitutes a study of significant clinical importance. By contrasting the work of Menzies and others, our research not only updates the theoretical contributions in this field but also provides a comprehensive evaluation of treatment methods for DA from a novel and focused clinical perspective. This unique viewpoint fills a gap in the existing research, underscoring the importance of an integrated approach to addressing patients’ death anxiety.

## 2 Materials and methods

### 2.1 Study design

The investigators performed an exhaustive search of five electronic databases (PubMed, EMBASE, the Cochrane Central Register of Controlled Trials, Web of Science, and Scopus) covering the period from their establishment to December 2023. The search strategy was carefully crafted using the PICOS framework: (P) Population: individuals experiencing DA; (I) Intervention: psychosocial interventions.; (C) Comparator: control group (CON) receiving only usual care; (O) Outcomes: assessment using the scale for DA testing only; (S) study type: RCTs. Exemplified using PubMed, the strategy is provided in Table 1.

**Table 1:** Approaches for study retrieval based on PubMed.

Search	PubMed
#1	Search: Psychosocial Intervention [MeSH Terms]
#2	Search: (((((((((Intervention, Psychosocial [Title/Abstract]) OR (Interventions, Psychosocial [Title/Abstract])) OR (Psychosocial Interventions [Title/Abstract])) OR (Psychological Intervention [Title/Abstract])) OR (Intervention, Psychological [Title/Abstract])) OR (Interventions, Psychological [Title/Abstract])) OR (Psychological Interventions [Title/Abstract])) OR (Psychological therapy [Title/Abstract])) OR (therapy [Title/Abstract])) OR (intervention [Title/Abstract])
#3	Search: (#1) OR (#2)
#4	Search: (death anxiety [Title/Abstract]) OR (Anxiety About Death [Title/Abstract])
#5	Search: (#1) OR (#2)
#6	Search: (#3) AND (#4)

### 2.2 Criteria for enrolling required articles

(1) Experimental group receiving various psychosocial therapies targeting DA in patients; (2) CON undergoing routine care; (3) conducted as a clinical RCT; (4) outcome indicators encompassing any of following items: Templer’s Death Anxiety Scale (TDAS), Death Anxiety Questionnaire (DAQ), or Templer Death Anxiety (TDA).

### 2.3 Criteria for excluding relevant articles

(1) Documents with missing or undisclosed data; (2) non-randomized controlled trials (RCTs) encompassing quasi-RCTs, animal studies, protocols, conference abstracts, case reports, or correspondence; and (3) assessing positive (acceptance subscale) and negative (fear and avoidance subscale) attitudes toward death simultaneously.

### 2.4 Study selection

A thorough screening and exclusion process was conducted using Zotero. To guarantee the highest evidence quality and minimize bias, only randomized controlled trials (RCTs) were included in this meta-analysis. This decision was informed by the need to rely on studies with robust methodological designs. Initially, two researchers identified and eliminated duplicates, non-randomized controlled trials (RCTs), review papers, conference papers, protocols, and communications based on the titles of the literature. Subsequently, researchers evaluated the abstracts of the literature to make decisions regarding inclusion and exclusion. The entire body of the remaining material was thoroughly examined by both researchers before making final selections. This process involved independent reviews by both researchers, who then compared their findings to ensure consistency. In instances of discrepancies, a third researcher was consulted to discuss and resolve the matter.

### 2.5 Data extraction

This research gathered data through utilizing a standardized seven-item data extraction form, data categories included (1) author; (2) publication year; (3) nation, to explore potential cross-cultural effects; (4) research period; (5) sample size; (6) mean age, to consider age-related effects on interventions; and (7) details of the psychological intervention.

### 2.6 Risk of bias (ROB) of encompassed literature

Following the Cochrane Handbook version 5.1.0 technique, two researchers independently appraised the ROB in these RCTs. The evaluation considered seven domains associated with bias: (1) random sequence generation, (2) allocation concealment, (3) blinding of participants and personnel, (4) blinding of outcome assessment, (5) incomplete outcome data, (6) selective reporting, and (7) other ROB. Depending on the number of components with potentially high ROB, RCTs were classified into high ROB (≥5), moderate ROB (3–4), and low ROB (≤2) (Higgins et al., 2011).

### 2.7 Data analysis

In this research using psychosocial therapies, all variables were of a continuous nature and would be displayed as means accompanied by standard deviation (SD). The presentation of continuous variables utilized standardized mean difference (SMD), calculated as the mean difference in outcomes between different groups divided by the SD of outcomes among subjects. This approach is particularly beneficial for aggregating data during trials with varying scales. The data were reported with 95% confidence intervals (CI). For our analytical approach, we opted for a random-effects model over a fixed-effects model. This decision was based on the recognition of potential variations among studies (Jackson et al., 2011).

This research employed Stata (version 15.1) following the PRISMA NMA instruction manual. The NMA aggregation and analysis were carried out using Markov chain Monte Carlo simulation chains within a Bayesian-based framework (Moher et al., 2015; Vats et al., 2019). In order to measure and illustrate concurrence between indirect and direct comparisons, the nodal method, as per relevant guidelines in State, was applied. The consistency test was considered successful if the *p* > 0.05 (Salanti et al., 2011). The network diagrams illustrating various psychosocial interventions were generated using Stata software. In these diagrams, connections among nodes symbolize direct comparisons made between various interventions. Each node in the network diagrams corresponds to a distinct psychosocial intervention or control condition. The width of the connecting lines and the dimensions of each node are scaled according to the quantity of studies (Chaimani et al., 2013). To summarize and depict the intervention hierarchy, the P score was employed, serving as a frequentist analog to surface under the cumulative ranking curve (SUCRA) values. Meanwhile, P score reflected the average certainty level indicating superiority of one treatment over another, as assessed across all competing treatments. This score can be 0–1, with 1 and 0 signifying the best and worst treatment, respectively. While P scores or SUCRA values can be presented as a percentage to convey the effectiveness or acceptability of psychosocial interventions, caution is advised in interpretation unless there are clinically meaningful differences between interventions (Marotta et al., 2020). To examine the potential bias caused by small-scale research, leading to publication bias (PB) in NMA, a network funnel plot was visually inspected using the symmetry criterion (Khera et al., 2016).

## 3 Results

### 3.1 Procedures for study screening and inclusion

**Figure 1:**
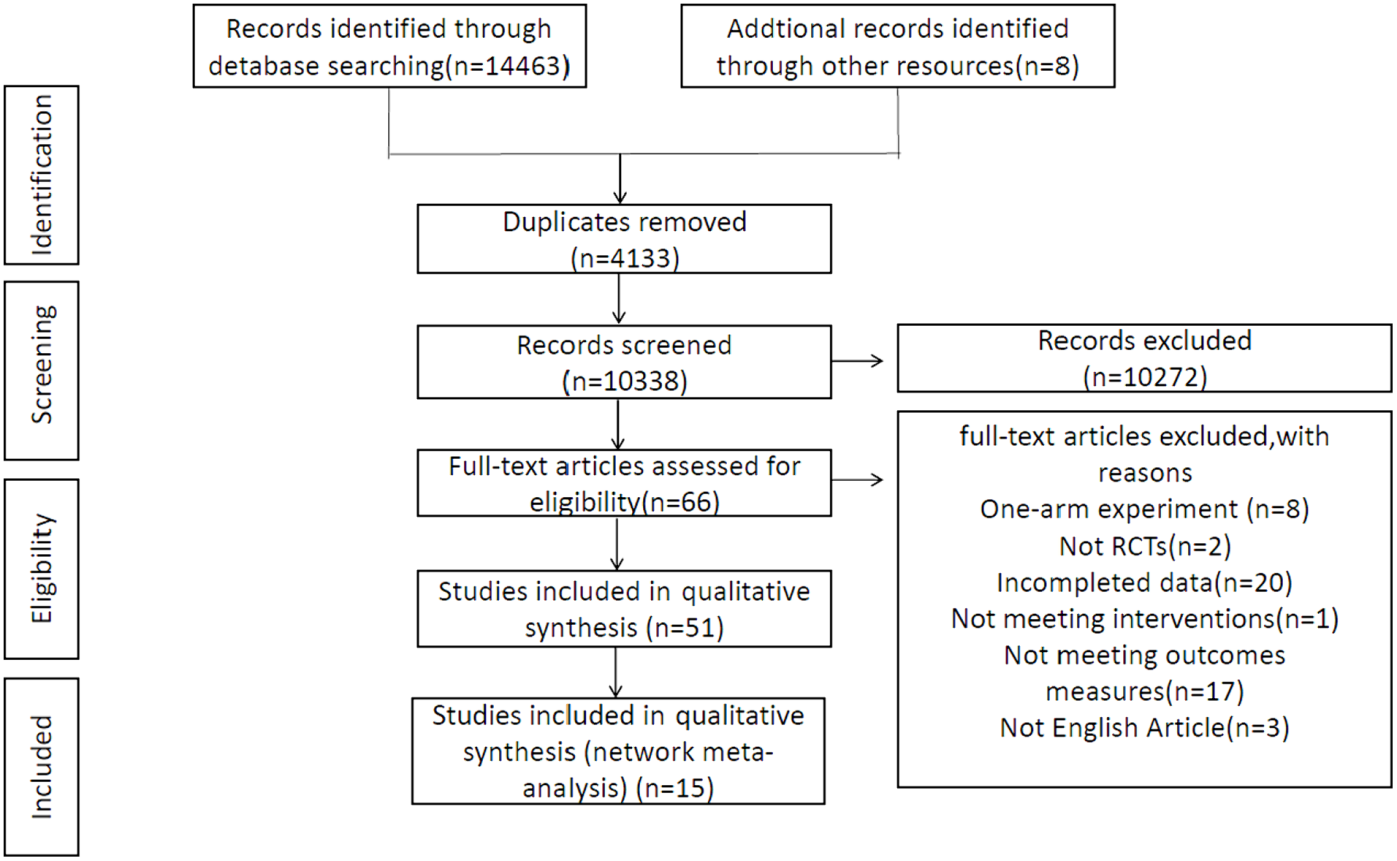
Procedures for literature inclusion.

Fourteen thousand four hundred sixty three studies were initially screened, and another eight articles were identified through manual searches. Upon elimination of duplicates, the remaining 10,338 documents underwent further scrutiny based on titles and abstracts, leading to the exclusion of additional documents. Subsequently, the 66 remaining ones experienced full-text review, further excluding 51 documents. The exclusion criteria included one-arm experiments, failure to align with the specified outcome measures, non-RCTs, incomplete data, not satisfying the interventions mentioned, and non-English articles. This meticulous selection process aimed at ensuring the inclusion of high-quality RCTs, thereby enhancing the reliability and applicability of our findings across different patient populations and settings. This process ultimately yielded a final set of 15 documents for inclusion in this research. Details were provided in Figure 1.

### 3.2 Quality of the enrolled studies

**Figure 2:**
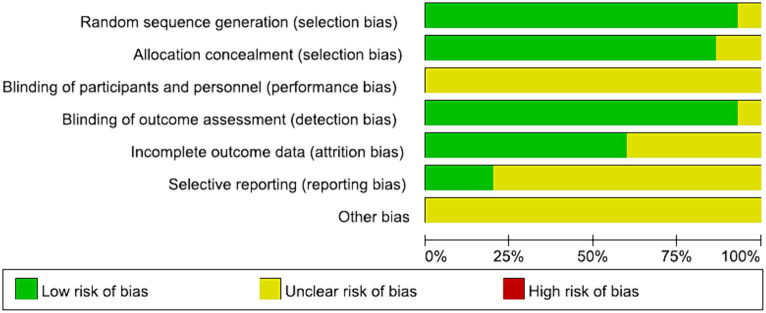
Graph for ROB.

**Figure 3:**
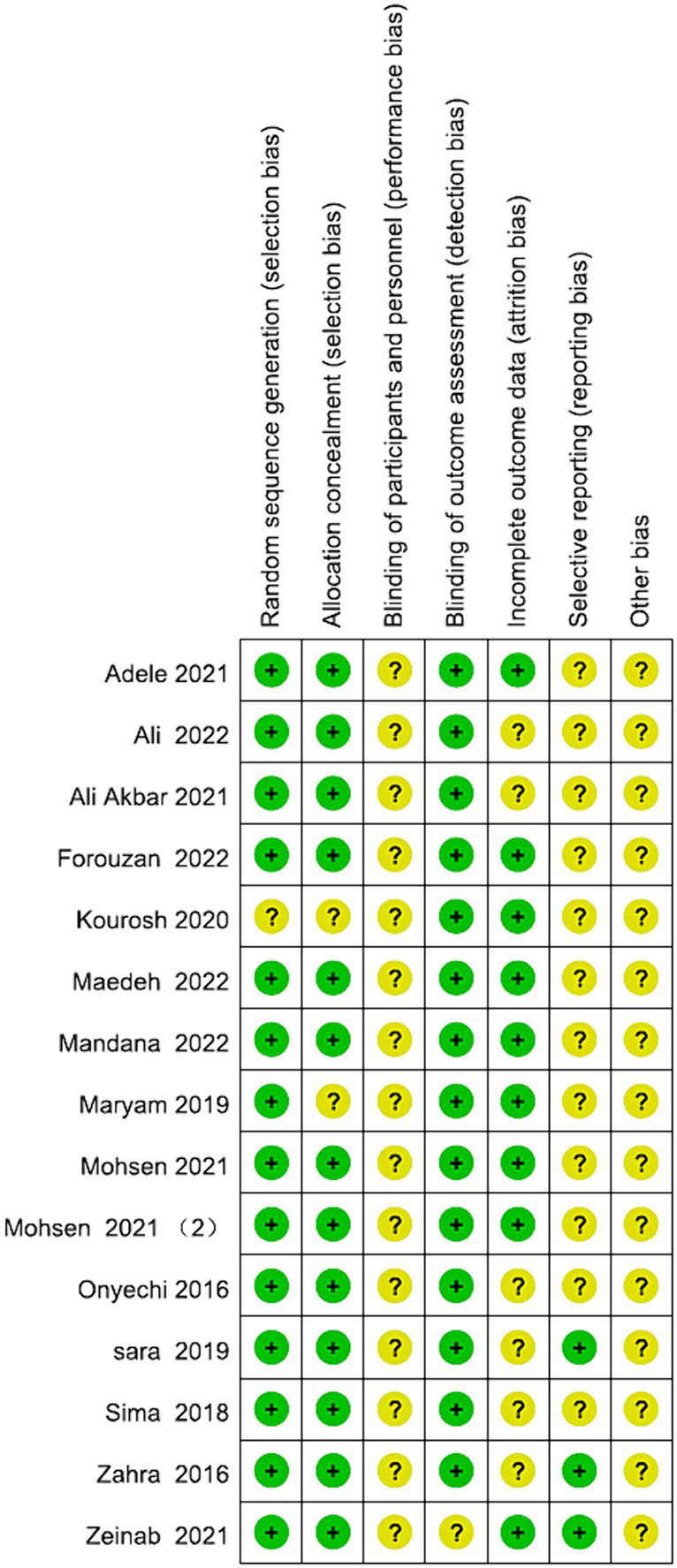
Summary of ROB.

All the studies were categorized as low risk. Simultaneous blinding of both subjects and measurers was not attained in any of these studies. Blinding challenges in psychosocial therapy trials highlight the complexity of conducting and evaluating interventions that inherently involve patient and therapist awareness of the treatment being administered. This lack of simultaneous is attributed to the nature of the intervention, which involved psychosocial therapies. Achieving simultaneous blinding of subjects and measurers proved challenging due to the necessity for both patients and their relatives to sign an informed consent prior to the experiment. This aspect was elucidated in Figures 2, 3.

### 3.3 Brief introduction of enrolled studies

**Figure 4:**
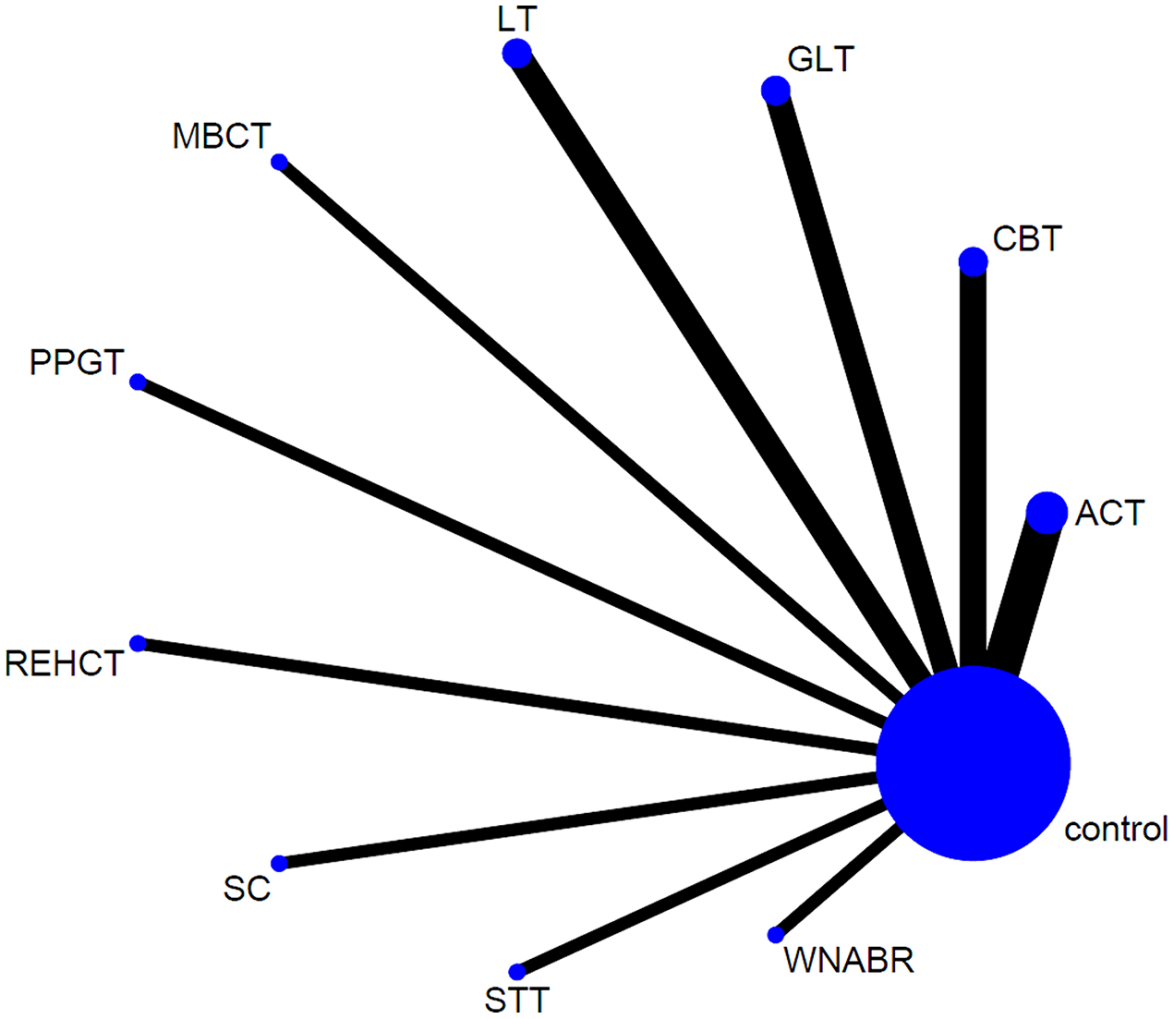
The full NMA figure.

To address the diversity of psychosocial interventions for death anxiety, our meta-analysis included a wide range of therapeutic approaches, reflecting the current scope of evidence-based practices in this area. Our analysis encompassed 15 RCTs in total, involving a cohort of 926 patients. The interventions in experimental group comprised the following: spiritual care (1 study) (Amini et al., 2020), group logotherapy (2 studies) (Emafti et al., 2019; Heidary et al., 2023), acceptance and commitment therapy (3 studies) (Farahi and Khalatbari, 2019; Sahebanmaleki et al., 2021; Fadhil et al., 2022), cognitive-behavioral therapy (2 studies) (Moradi et al., 2022; Saki et al., 2022), logotherapy (2 studies) (Akbari et al., 2021; Bahar et al., 2021), rational-emotive hospice care therapy (1 study) (Onyechi et al., 2016), spirituality therapy training (1 study) (Ameri et al., 2021), positive psychology group therapy (1 study) (Saeedi et al., 2016), mindfulness based cognitive therapy (1 study) (Nabipour et al., 2018), and white noise and Benson’s relaxation technique (1 study) (Ahmadi et al., 2022). Thirteen studies utilized TDAS as an outcome indicator. TDAS consists of 15 items, where correct in 9 items are scored 1, and incorrect responses in 6 items are also scored 1. The total score is 0–15, with a higher score reflecting increased death anxiety. One study utilized the TDA severity subscale, which comprises 25 items (17 positive and 8 negative) with a 5-point Likert scale ranging from never to very high, formed and verified by Templer in 1970 (Templer, 1970). Another study employed the DAQ, an Igbo version of a 15-item questionnaire from Conte’s DA questionnaire (Conte et al., 1982) to judge the extent of DA experienced by cancer patients and their caregivers. Table 2 provides detailed attributes of the RCTs incorporated in the meta-analysis (Figure 4).

**Table 2:** Brief introduction of enrolled studies.

Author	Country	Year	Sample size	Intervention	Outcome
Ali et al.	Iran	2021	120	SC	T-DAS
Zeinab et al.	Iran	2021	30	STT	T-DAS
Kourosh et al.	Iran	2020	145	SC	T-DAS
Adele et al.	Iran	2021	40	LT	TDA severity subscale
Maryam et al.	Iran	2019	59	GLT	T-DAS
Ali et al.	Iraq	2022	70	ACT	T-DAS
Sara et al.	Iran	2019	30	ACT	T-DAS
Maedeh et al.	Canada	2022	63	GLT	T-DAS
Mohsen et al.	Iran	2019	66	CBT	T-DAS
Forouzan et al.	Iran	2022	60	WNABR	T-DAS
Sima et al.	Iran	2018	30	MBCT	T-DAS
Onyechi et al.	Nigeria	2016	32	REHCT	DAQ
Zahra et al.	Iran	2016	32	PPGT	T-DAS
Mohsen et al.	Iran	2021	24	ACT	T-DAS
Mohsen et al.	Iran	2021	66	CBT	T-DAS

### 3.4 Network meta-analysis

#### 3.4.1 Unified death anxiety rating scale

Consistency and inconsistency of all studies were evaluated by testing *p*-values for comparisons. In all cases, the obtained *p*-values exceeded 0.05, affirming the acceptability of consistency across studies in terms of their effects. Further details are provided in Table 3.

**Table 3:** League table on death anxiety.

Comparison	Mean difference (95% CI)	Comparison	Mean difference (95% CI)
REHCT vs. LT	40.77 (36.74,44.79)	LT vs. PPGT	−2.47 (−5.29,0.35)
REHCT vs. STT	41.28 (37.12,45.44)	STT vs. MBCT	−1.65 (−4.65,1.35)
LT vs. STT	0.51 (−2.24,3.27)	STT vs. CBT	−1.66 (−4.16,0.83)
LT vs. GLT	−0.60 (−3.01,1.81)	STT vs. PPGT	−1.96 (−4.97,1.05)
LT vs. ACT	−0.97 (−3.11,1.16)	GLT vs. MBCT	−1.56 (−4.24,1.11)
LT vs. WNABR	−1.66 (−4.24,0.92)	GLT vs. CBT	−1.58 (−3.67,0.52)
STT vs. GLT	−0.09 (−2.71,2.53)	GLT vs. PPGT	−1.87 (−4.56,0.82)
STT vs. ACT	−0.46 (−2.84,1.92)	ACT vs. MBCT	−1.19 (−3.63,1.25)
STT vs. WNABR	−1.15 (−3.94,1.64)	ACT vs. CBT	−1.20 (−2.99,0.58)
GLT vs. ACT	−0.37 (−2.33,1.58)	ACT vs. PPGT	−1.50 (−3.96,0.96)
GLT vs. WNABR	−1.06 (−3.50,1.37)	WNABR vs. MBCT	−0.50 (−3.34,2.34)
ACT vs. WNABR	−0.69 (−2.87,1.49)	WNABR vs. CBT	−0.51 (−2.82,1.79)
REHCT vs. GLT	41.37 (37.44,45.30)	WNABR vs. PPGT	−0.81 (−3.66,2.04)
REHCT vs. ACT	41.74 (37.97,45.51)	MBCT vs. CBT	0.01 (−2.54,2.57)
REHCT vs. WNABR	42.43 (38.38,46.47)	MBCT vs. PPGT	−0.31 (−3.37,2.75)
REHCT vs. MBCT	42.93 (38.74,47.12)	CBT vs. PPGT	−0.30 (−2.87,2.27)
REHCT vs. CBT	42.94 (39.09,46.79)	REHCT vs. SC	44.56 (40.52, 48.60)
REHCT vs. PPGT	43.24 (39.04,47.44)	LT vs. SC	−3.79 (−6.37, −1.22)
REHCT vs. Control	−44.88 (−48.47, −41.28)	CBT vs. Control	−1.94 (−3.31, −0.57)
LT vs. MBCT	−2.16 (−4.97,0.64)	STT vs. SC	−3.28 (−6.06, −0.50)
LT vs. CBT	−2.18 (−4.44,0.08)	GLT vs. SC	−3.19 (−5.62, −0.76)
CBT vs. SC	−1.62 (−3.91,0.68)	ACT vs. SC	−2.82 (−4.99, −0.65)
PPGT vs. SC	−1.32 (−4.17,1.53)	WNABR vs. SC	−2.13 (−4.74,0.48)
SC vs. Control	0.32 (−1.52,2.16)	MBCT vs. SC	−1.63 (−4.46,1.20)
LT vs. Control	−4.11 (−5.91, −2.32)	STT vs. Control	−3.60 (−5.69, −1.51)
GLT vs. Control	−3.51 (−5.10, −1.93)	ACT vs. Control	−3.14 (−4.28, −1.99)
WNABR vs. Control	−2.45 (−4.30, −0.60)	PPGT vs. Control	−1.64 (−3.81,0.53)

SC, spiritual care; GLT, group logotherapy; ACT, acceptance and commitment therapy; CBT, cognitive-behavioral therapy; LT, logotherapy; STT, spirituality therapy training; REHCT, rational-emotive hospice care therapy; PPGT, positive psychology group therapy; MBCT, mindfulness based cognitive therapy; WNABR, white noise and Benson’s relaxation technique.

**Figure 5:**
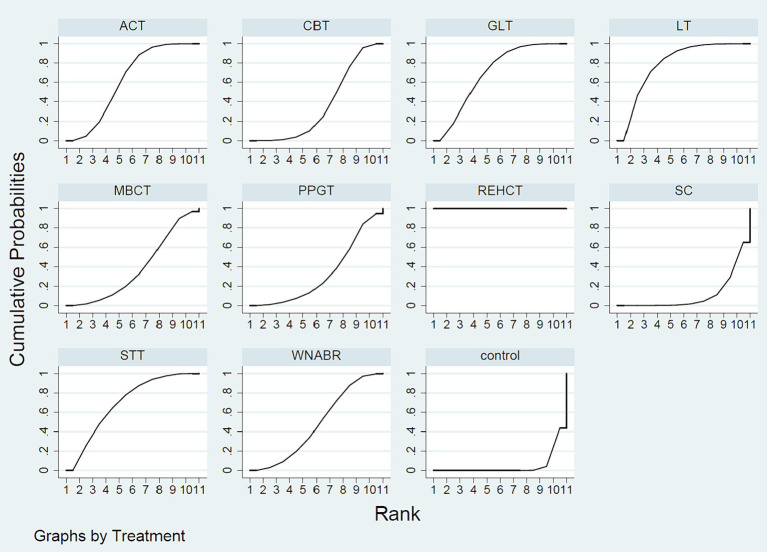
SUCRA plot.

Evaluating the effectiveness of various psychosocial interventions provides critical insights into their relative efficacy, guiding clinicians in selecting the most appropriate treatment based on empirical evidence. The NMA outcomes demonstrated that in contrast to routine interventions, rational-emotive hospice care therapy [Mean Difference (MD) = −44.88, 95% CI = (−48.47, −41.28)], logotherapy [MD = −4.11, 95% CI = (−5.91, −2.32)], spirituality therapy training [MD = −3.60, 95% CI = (−5.69, −1.51)], group logotherapy [MD = −3.51, 95% CI = (−5.10, −1.93)], acceptance and commitment therapy [MD = −3.14, 95% CI = (−4.28, −1.99)], white noise and Benson’s relaxation technique [MD = −2.45, 95% CI = (−4.30, −0.60)], cognitive-behavioral therapy [MD = −1.94, 95% CI = (−3.31, −0.57)] were all superior in reducing the DA scores. The NMA findings further revealed that in comparison to rational-emotive hospice care therapy, logotherapy [MD = 40.77, 95% CI = (36.74, 44.79)], spirituality therapy training [MD = 41.28, 95% CI = (37.12, 45.44)], group logotherapy [MD = 41.37, 95% CI = (37.44, 45.30)], acceptance and commitment therapy [MD = 41.74, 95% CI = (37.97, 45.51)], white noise and Benson’s relaxation technique [MD = 42.43, 95% CI = (38.38, 46.47)], mindfulness based cognitive therapy [MD = 42.93, 95% CI = (38.74, 47.12)], cognitive-behavioral therapy [MD = 42.94, 95% CI = (39.09, 46.79)], positive psychology group therapy [MD = 43.24, 95% CI = (39.04, 47.44)], spiritual care [MD = 44.56, 95% CI = (40.52, 48.60)] where all less effective in reducing DA of patients. Comparatively, to spiritual care, logotherapy [MD = −3.79, 95% CI = (−6.37, −1.22)], spirituality therapy training [MD = −3.28, 95% CI = (−6.06, −0.50)], group logotherapy [MD = −3.19, 95% CI = (−5.62, −0.76)], acceptance and commitment therapy [MD = −2.82, 95% CI = (−4.99, −0.65)] exhibited higher performance in DA reduction. The likelihood-based ranking of various interventions in reducing scores was topped by rational-emotive hospice care therapy (with a SUCRA of 100%, as depicted in Figure 5).

### 3.5 PB test

**Figure 6:**
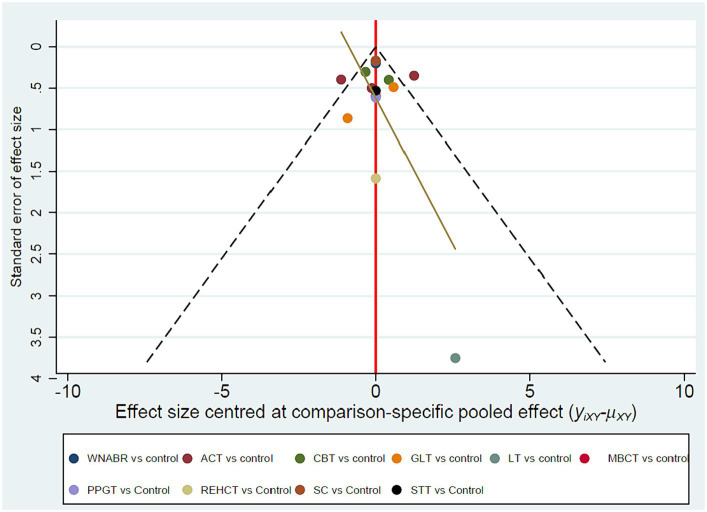
Funnel plots.

Distinct funnel plots for all outcome indicators were generated to assess potential PB upon visual inspection, these funnel plots failed to reveal any remarkable PB, as explicated in Figure 6.

## 4 Discussion

This research intended to assess and compare the roles of various psychological therapies in alleviating DA of patients by comprehensively reviewing 15 distinct psychological treatments on 926 patients. The sample size is deemed substantial in the context of the study. Our research demonstrated the best performance of rational-emotive hospice care therapy in decreasing DA of patients.

Therapists employ REBT theory, formulated by Albert Ellis in 1955, to address human disturbance issues. The fundamental premise of rational-emotive hospice care therapy is rooted in the concept that individuals largely disturb themselves, implying that, with therapeutic assistance, they can proactively take steps to alleviate such disturbance (Ellis, 1957, 1962, 1989). As highlighted by Ellis and Bernard (1985), practitioners of rational emotive behavior therapy guide individuals in recognizing how their attitudes and beliefs contribute to emotional distress and interpersonal challenges, fostering a sense of control over their destiny. The rational-emotive hospice care therapy is constructed on a cognitive-behavioral therapy approach, combining quotes from Albert Ellis to facilitate the challenging of problematic assumptions of participants and encourage a transformation in their dysfunctional emotions and thoughts related to cancer. Recognizing the potential reluctance of many cancer patients, particularly those highly distressed, the researchers incorporated Sobel’s seven-step decision-making process into the rational-emotive hospice care therapy manual to facilitate informed decision-making in addressing detrimental emotions (Woods, 1981). This sequential decision-making process focuses on individual concerns one at a time. As patients engage with and resolve consecutive concerns, denial diminishes, and a renewed determination to live, even in the face of impending death, emerges. The steps encompass: identifying primary emotions, delineating the most significant problems and associated issues; creating alternatives and discerning covert structures; imagining the mechanism for others to respond to similar problems; weighing the advantages and disadvantages of each proposed solution; prioritizing all potential solutions; opting for the most viable solution; and reevaluating and redefining primary challenges based on the assessment.

Furthermore, the NMA results signified that in addition to the rational-emotive hospice care therapy, group logotherapy, logotherapy, spirituality therapy training, and acceptance and commitment therapy emerged as the most effective psychotherapies for addressing DA of patients. These therapies ranked within the top five in terms of effectiveness for DA reduction. Logotherapy, described as a therapeutic approach centered on meaning, aligning with the principles of cognitive-behavioral therapy, seeks to enhance the efficacy of the therapeutic process (Ameli and Dattilio, 2013). Group logotherapy, rooted in logotherapy principles, addresses personal experiences of existential isolation within a group setting. By collectively creating a collectively understood significance of shared adversity, group logotherapy alleviates the sense of isolation, fostering a connection among group members. Additionally, group logotherapy leverages the therapeutic value of exploring existential issues embedded in the group process (Somov, 2007). However, this research affirmed no remarkable difference between two therapies in reducing patients’ DA. It is plausible that finding the meaning one’s life is more intricately linked to the individual themselves. Several studies emphasize the impact of spiritual therapy in reducing cognitive avoidance and psychological distress among the elderly (Ameli and Dattilio, 2013) and improving spiritual health and quality of life in cancer patients (Somov, 2007; Menzies et al., 2018; Li et al., 2021). Acceptance and commitment therapy, grounded in a philosophy that eschews creating a reality-matching model, emphasizes accuracy, breadth, and depth in predicting and changing behavior. Acceptance and commitment therapy contributes to improved quality of life, heightened creativity, reduced risk of job burnout, and enhanced learning abilities (Li et al., 2021). Considering these advantages and theoretical foundations, we hypothesized that combining logotherapy and spirituality therapy training or acceptance and commitment therapy would yield a more favorable effect on DA than employing rational emotive hospital care therapy alone. In the future, the invention of a therapy effectively amalgamating these advantages holds the potential to become a widely embraced and enduring psychological intervention for reducing the death anxiety in patients. However, further studies are imperative to substantiate our hypothesis.

## 5 Strengths and shortcomings

Firstly, this is the inaugural NMA comparing various therapies to reduce the DA in patients, a topic not previously explored by scholars. Prior meta-analyses focused on psychotherapy effects concerning patient attitudes toward death, including acceptance of death and DA (Menzies et al., 2018). The outcome measure of this research specifically addressed the patient’s fear of death.

Secondly, the analytical method employed in this research is highly rigorous. To assess the quality, each article was jointly evaluated by two scholars. In the event of disagreement, three scholars collaborated in the assessment process. Simultaneously, we maximized the inclusion of literature, resulting in generally high-quality final selections. Consequently, our evidence is notably comprehensive. Overall, this is a clinically significant study.

Finally, readers should carefully interpret our research results. Because the number of articles included is small, with only 15 studies, which blocks the data analysis based on confounding factors such as duration of intervention, frequency of intervention, duration of each intervention, age proportion of patients, and logarithmic sex ratio of patients. While acknowledging the limitations of this review, including the focus on RCTs and specific interventions, future research directions are proposed to explore the efficacy of a broader range of therapeutic approaches and to investigate the role of cultural, age-related, and gender differences in the treatment of death anxiety.

## 6 Conclusion

This research demonstrated rational-emotive hospice care therapy as the optimal psychosocial intervention in decreasing the DA in patients. However, more relevant RCTs characterized by high-quality, rigorous experimental methods, and longer follow-up are essential to validate this viewpoint.

## Data availability statement

The original contributions presented in the study are included in the article/supplementary material, further inquiries can be directed to the corresponding author.

## Author contributions

JL: Conceptualization, Data curation, Formal analysis, Funding acquisition, Investigation, Methodology, Project administration, Resources, Software, Supervision, Validation, Visualization, Writing – original draft, Writing – review & editing. YY: Data curation, Investigation, Writing – review & editing. HC: Data curation, Software, Writing – review & editing. HM: Formal analysis, Writing – review & editing. YT: Formal analysis, Writing – review & editing.
